# 

**Published:** 1895-06

**Authors:** 


*PRINTERS' INK*, OF MAY 8, 1895, SAYS THIS JOURNAL HAS THE LARGEST CIRCULATION OF ANY GEORGIA MEDICAL JOURNAL. THAT IS TRUE.

ESTABLISHED 1855. SUBSCRIPTION, $2.00.

### ATLANTA MEDICAL AND SURGICAL JOURNAL.

LUTHER B. GRANDY, M.D., MANAGING EDITOR.

M. B. HUTCHINS, M.D., BUSINESS MANAGER.

COLLABORATORS:

H. V. M. MILLER, M.D., LL.D., VIRGIL O. HARDON, M.D., FLOYD W. McRAE, M.D., DUNBAR ROY, M.D., AND H. R. SLACK, M.D., PH.M.

VOLUME XII. NEW SERIES. ATLANTA, GA., JUNE, 1895. No. 4.

MERCAURO

MERCAURO
MERCAURO
MERCAURO
MERCAURO

THE IDEAL ALTERATIVE

NO STOMACHIC DISTURBANCE

MERCAURO
MERCAURO
MERCAURO
MERCAURO

MERCAURO

CHAS. ROOME PARMELE CO., 98 WILLIAM ST., N. Y.

PUBLISHED BY THE FRANKLIN PRINTING AND PUBLISHING CO., ATLANTA, GA.

Entered at the Post-Office at Atlanta, Ga., as second-class matter.

